# Editorial: Neuroimmune Interactions in Peripheral Neuropathy

**DOI:** 10.3389/fnmol.2022.929081

**Published:** 2022-05-23

**Authors:** Jing Yang, Avraham Yaron, Kai Liu

**Affiliations:** ^1^School of Life Sciences, Peking University, Beijing, China; ^2^Department of Biomolecular Sciences, Weizmann Institute of Science, Rehovot, Israel; ^3^Department of Molecular Neuroscience, Weizmann Institute of Science, Rehovot, Israel; ^4^Division of Life Science, The Hong Kong University of Science and Technology, Clear Water Bay, Hong Kong SAR, China

**Keywords:** neuroimmune, peripheral neuropathy, axon degeneration, inflammation, immune cells

Peripheral neuropathy is a collection of neurodegenerative conditions in the peripheral nervous system (PNS), particularly involving damage or loss of sensory, motor, or autonomic axons. In the past decade, there have been tremendous advances in understanding the mechanisms of such neuropathic events in the PNS, including those encountered in traumatic nerve injuries, chemotherapy-induced peripheral neuropathy, diabetic peripheral neuropathy, and many other neurological diseases (Coleman and Hoke, 2020; Figley and DiAntonio, 2020; Sambashivan and Freeman, 2021). In addition, peripheral neuropathy in “unconventional” scenarios has been identified in different organs, such as the gastrointestinal tract, pancreas, and liver (Alvarsson et al., 2020; Liu et al., 2021; Sun et al., 2021). These scientific discoveries have significantly promoted our knowledge of neurodegeneration and its relationship to other systems in the body.

Numerous studies have documented the extensive communication and interaction between the nervous and immune systems. It has long been known that neuropathic events would trigger profound inflammatory responses, e.g., glial activation during neurodegeneration in the central nervous system or immune cell recruitment in a damaged peripheral nerve. Such neuroimmune interactions represent an indispensable part of disease manifestations and may directly contribute to neural damage or repair processes (Kiefer et al., 2001; Said, 2007; Glass et al., 2010; Ransohoff, 2016). Therefore, exploring neuroimmune mechanisms of peripheral neuropathy has become an exciting frontier of the research field.

In the Brief Research Report, Zhang et al. reported how degenerating axons would trigger the recruitment of macrophages in traumatically-injured mouse sciatic nerves. In particular, they observed the upregulation of ADP-dependent glucokinase (ADPGK) in macrophages locally accumulated in the injured nerve segments. Of importance, ADPGK could promote the phagocytotic activity of those macrophages, thus likely facilitating the clearance of axonal debris in this context of peripheral neuropathy.

The recent development of 3D imaging techniques has enabled the comprehensive, accurate assessment of neural structures in various intact tissues (Tainaka et al., 2016; Ueda et al., 2020; Richardson et al., 2021). In the Original Research article, Hu et al. exploited such advanced imaging power and reported the 3D anatomy of autonomic innervations in the mouse and human prostates for the first time. Moreover, the authors uncovered that loss of local sympathetic axons in the mouse prostate would cause the sterile inflammation mimicking the disease condition of chronic non-bacterial prostatitis/chronic pelvic pain syndrome. Mechanistically, the sympathetic signal might directly control the inflammatory response of macrophages in the prostate. Those findings have established a new, previously-unrecognized scenario of peripheral neuropathy and elucidated its importance to the neuroimmune interaction in this specific prostate disease.

While peripheral neuropathy could actively elicit inflammatory responses, the immune system also influences different neural functions. In the Review article, Gao et al. summarized the current evidence supporting that chronic inflammation occurring in the peripheral demyelinating polyneuropathy would impair the structural and functional integrity of the nodes of Ranvier. Such immune action might contribute to the malfunction of the myelin sheath and thus lead to the disruption of neural signals. The authors further highlighted the relevance of this neuroimmune interplay to the disease progression and the development of therapeutic strategies.

Although neuroimmune aspects of peripheral neuropathy have increasingly garnered attention in the past years, critical details of pathological mechanisms remain to be fully charted out. In the Perspective article, Zhou et al. discussed the functional link of chronic systemic inflammation to the onset of neuropathic pain. In particular, the authors pointed out that research has been mainly focusing on the local inflammation in neural tissues such as the spinal cord but tended to neglect the potential role of inflammatory cues broadly present in the body. The authors called for a more integrated view of local and systemic effects of inflammation during peripheral neuropathy and their synergetic roles in chronic pain.

These articles in this Research Topic represent the emerging frontier of neuroimmune interactions in peripheral neuropathy. It has become evident that the nervous system and its neuropathic events would influence tissue immunity *via* divergent signaling mechanisms. At the same time, immune responses, either locally or systemically, could impinge on the physiology and homeostasis of the nervous system. Therefore, it is essential to emphasize this bi-directional neuroimmune crosstalk in any disease context. Still, many questions await future inquiries in the field. Continuous research exploring the significance and complexity of neuroimmune interactions in peripheral neuropathy would advance the in-depth knowledge of disease manifestations. Undoubtedly, such efforts hold the promise of identifying novel entry points for conquering those dreadful, debilitating neurological conditions.

## Author Contributions

All authors listed have made a substantial, direct, and intellectual contribution to the work and approved it for publication.

## Conflict of Interest

The authors declare that the research was conducted in the absence of any commercial or financial relationships that could be construed as a potential conflict of interest.

## Publisher's Note

All claims expressed in this article are solely those of the authors and do not necessarily represent those of their affiliated organizations, or those of the publisher, the editors and the reviewers. Any product that may be evaluated in this article, or claim that may be made by its manufacturer, is not guaranteed or endorsed by the publisher.
